# Towards green extraction of bioactive natural compounds

**DOI:** 10.1007/s00216-023-04969-0

**Published:** 2023-10-03

**Authors:** Miguel Herrero

**Affiliations:** Laboratory of Foodomics, Institute of Food Science Research–CIAL (CSIC-UAM), Calle Nicolás Cabrera 9, 28049 Madrid, Spain

**Keywords:** Bioactive compounds, Natural matrices, Agri-food by-products, Green extraction, Biorefinery

## Abstract

The increasing interest in natural bioactive compounds is pushing the development of new extraction processes that may allow their recovery from a variety of different natural matrices and biomasses. These processes are clearly sought to be more environmentally friendly than the conventional alternatives that have traditionally been used and are closely related to the 6 principles of green extraction of natural products. In this trend article, the most critical aspects regarding the current state of this topic are described, showing the different lines followed to make extraction processes greener, illustrated by relevant examples. These include the implementation of new extraction technologies, the research on new bio-based solvents, and the development of new sequential process and biorefinery approaches to produce a full valorization of the natural sources. Moreover, the future outlook in the field is presented, in which the main areas of evolution are identified and discussed.

**Graphical abstract:**

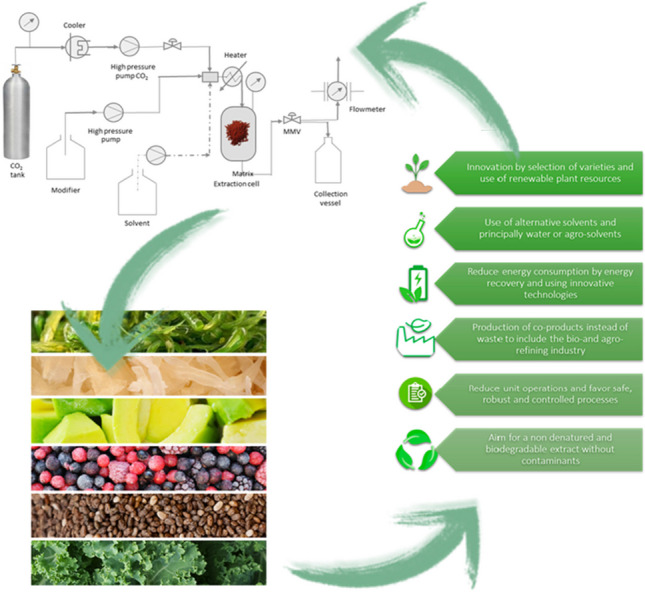

## Introduction

**Fig. 1:**
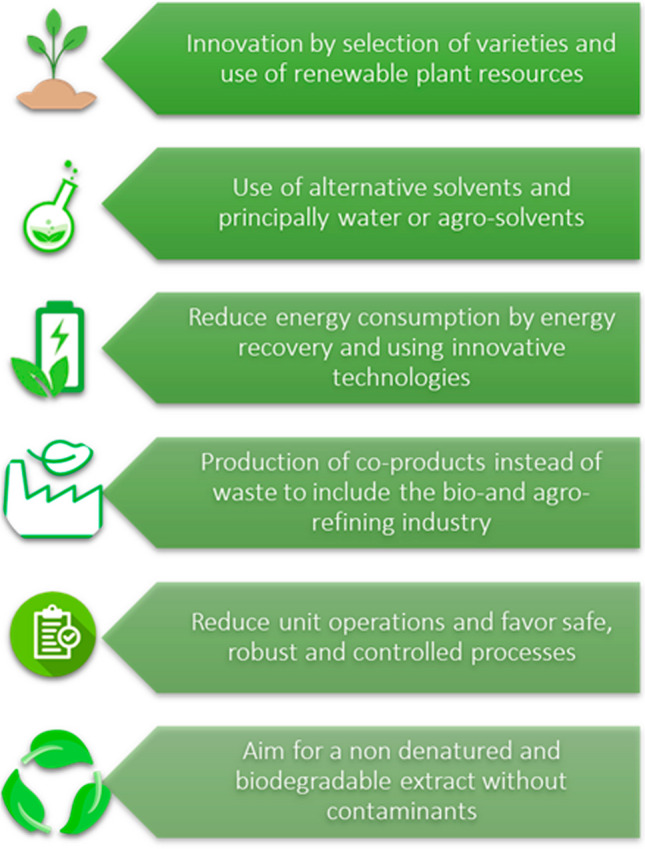
The six principles of green extraction of natural products, as proposed by Chemat et al. [7]

In spite of the efforts of some climate change negationists and global warming skepticism leaders behaving as if they were part of the film “Don’t look up” by Adam McKay, strong scientific evidence points to the effect of human activity on climate change [1]. This observation has even led to the definition of Anthropocene as a new geological epoch [2]. As we scientists build our work on facts and not on beliefs, efforts are being produced in every research field to reduce the impacts on the environment, and analytical chemistry is not an exception. The need for a more sustainable approach to research in Chemistry was already envisaged 25 years ago by Anastas and Warner who defined the 12 principles of Green Chemistry [3]. From that point, different milestones and principles have been developed for the practice of green analytical chemistry [4, 5], green sample preparation [6], and for the green extraction of natural products [7] (Fig. 1). This latest field of application is at the core of the present manuscript, and has experienced an exponential growth in the last years.

Besides the focus on more sustainable extraction tools for the recovery of natural bioactive compounds, the relevance of this field is also related to the target compounds. The food industry is demanding a new range of compounds and extracts from natural origin to address consumers’ needs and preferences related to the development of new products. Nowadays, the use of natural compounds is clearly preferred over the use of synthetic alternatives. Moreover, in a context in which purely nutritional needs are largely covered in the western countries, consumers move their attention to products that are able to provide an additional health benefit. The combination of these factors is pushing the search for new natural sources of bioactive compounds, both known, and novel promising compounds. Although the list of potentially natural bioactive compounds being investigated at present is huge, some groups of components that attract attention include polyphenols, carotenoids and other terpenoids, alkaloids, polyunsaturated fatty acids, or saponins, just to name a few. The variability in the chemical composition of the natural matrix as well as the level of purity desired will guide the selection and complexity of the extraction protocol.

In terms of natural samples, traditionally, biomasses rich in bioactive secondary metabolites, such as plants and seaweeds, have been widely studied [8]. However, in the last years, as a result of the push towards sustainability and to the development of circular economy-related approaches, agri-food by-products have raised significant attention. There is a wealth of research showing how some of these by-products that were traditionally underused or entirely disposed are still rich on bioactive compounds that can be valorized into added-value products [9]. Lastly, the use of microalgae as natural sources for bioactives is also very relevant, as these microorganisms can be cultured under specific conditions to overproduce particular compounds of interest [10]. Besides, non-arable land might be used for their production. Both photobioreactor technology and biotechnology advancements are fostering the commercial production of an ever increasing number of different strains [11].

All these factors are combined at present in the search for new green alternatives. In this context, the six principles of green extraction [7] are closely related to the change observed in the recently published applications and developments towards a more sustainable approach. In fact, all of them are linked to the current and future trends observed in the field of natural bioactives extraction. The present Trends article presents a succinct non-comprehensive overview highlighting the most relevant recent applications developed around these principles illustrating the most important advancements made in the field, as well as the future perspectives, developments, and research directions in the area.

## Current situation

As already mentioned, the current state within the extraction of bioactive compounds from natural matrices field is strongly marked for the development of new processes that may either improve the existing ones in terms of sustainability, greenness, and efficiency or that can be used to explore the possibilities for underused biomasses. The most successful advanced extraction techniques used in the field so far include microwaves-assisted extraction (MAE), ultrasounds-assisted extraction (UAE), the use of compressed fluids under different conditions (supercritical fluids extraction, SFE; pressurized liquids extraction, PLE; gas-expanded liquids, GXE), pulsed electric fields (PEF), and enzyme-assisted extraction (EAE). Table 1 shows a summary of the most notable features of these techniques. Other techniques have also been reported as being green and efficient, as those based on mechanochemical reactions or high-pressure homogenization.

**Table 1:** Summary of the most-used advanced green extraction techniques used at present for the recovery of bioactive compounds from natural samples

Technique	Typical extraction conditions	Advantages	Disadvantages	Target bioactives
Microwaves-assisted extraction — MAE	Power: 100–600 W Temp: 30–100 °C Press: 10–40 MPa Extraction time: 5–30 min	Fast extractions Good efficiency	Need of temperature control	Polyphenols Polysaccharides Carotenoids
Ultrasounds-assisted extraction — UAE	Frequency: 20–100 kHz Temp: 30–60 °C Extraction time: 15–80 min	No high temperatures needed Fast and cheap Variety of solvents	Non-modifiable polarity	Polyphenols Alkaloids
Supercritical fluid extraction —SFE	Temp: 35–60 °C Press: 10–40 MPa Extraction time: 60–180 min	Very selective Solvent-free extracts	Low affinity for polar compounds Costly equipment	Terpenes Carotenoids PUFAs
Pressurized liquids extraction — PLE	Temp: 50–200 °C Press: 5–15 MPa Extraction time: 5–30 min	Fast extractions Lower volumes of solvent needed High total extraction yields	Relatively lack of selectivity Possibility of degradation	Polyphenols Carbohydrates
Gas-expanded liquids extraction — GXL	Temp: 35–60 °C Press: 10–25 MPa Extraction time: 60–120 min	Selectivity modulated by expanded solvent Tunable solvent properties	Costly equipment More difficult process optimization	Carotenoids Fatty acids Terpenoids Polyphenols
Pulsed electric fields — PEF	Electric field strength: 0.5–30 kV/cm Pulse duration: 2–5 µs Number of pulses: 20–200	Lower energy consumption Higher extraction yields	Need of solvent-based recovery	Polyphenols Sterols Polysaccharides Carotenoids
Enzyme-assisted extraction — EAE	Temp: 30–50 °C Extraction time: 2–24 h	Improved recovery Mild conditions	Long process times Need of post-extraction treatments	Lipids Oils Polyphenols Carbohydrates

*Temp*, temperature; *press*, pressure; *PUFA*, polyunsaturated fatty acids

In this section, the most notable recent advances in the field are discussed and commented, highlighting the evolution observed in the last 10 years regarding the principles of green extraction, summarized in Fig. 1. Although shown separately, it has to be considered that many of the current approaches are closely related to several of those principles.

### Innovation by the selection of varieties and the use of renewable plant resources

This section represents one of the most important points in the last years. The study of new natural sources of bioactive compounds is continuously growing both to discover new interesting compounds and to gain advantage of underused biomasses. Among them, as it has been already mentioned, the use of agri-food by-products represents a subfield of special interest. The use of these materials allows not only the proper recovery of the bioactive components but also to generate added-value solutions from wastes, increasing the sustainability of the industrial processes as well as improving aspects related to circular economy [12]. The number of by-products studied so far is huge, being the applications reported related to several extraction tools [13, 14]. The scientific evidence pointing at their rich profile on bioactive compounds, in general, is increasing [15]. For instance, PLE has been used to recover bioactives from artichoke by-products [16], whereas UAE is one of the most extended advanced extraction methods for the recovery of a variety of different compounds, such as chlorophylls from tomato and carrot by-products [17].

Another remarkable example is the search for new organisms that could be grown for the production of bioactive compounds, such as microalgae or fungi. The use of green approaches, such as UAE, was demonstrated to provide with higher efficiency for the recovery of interesting compounds from *Scenedesmus obliquus* microalga, compared to conventional extractions [18]. Moreover, the use of SFE provided with a high selectivity towards the extraction of carotenoids with a high-purity extract. Another very interesting approach was followed for the extraction of bioactive compounds from *Tetradesmus obliquus* by SWE. In this case, the overall sustainability was even improved by the fact that the culture was developed using poultry wastewater as growth medium [19].

### Use of alternative solvents and bio-based solvents

**Fig. 2:**
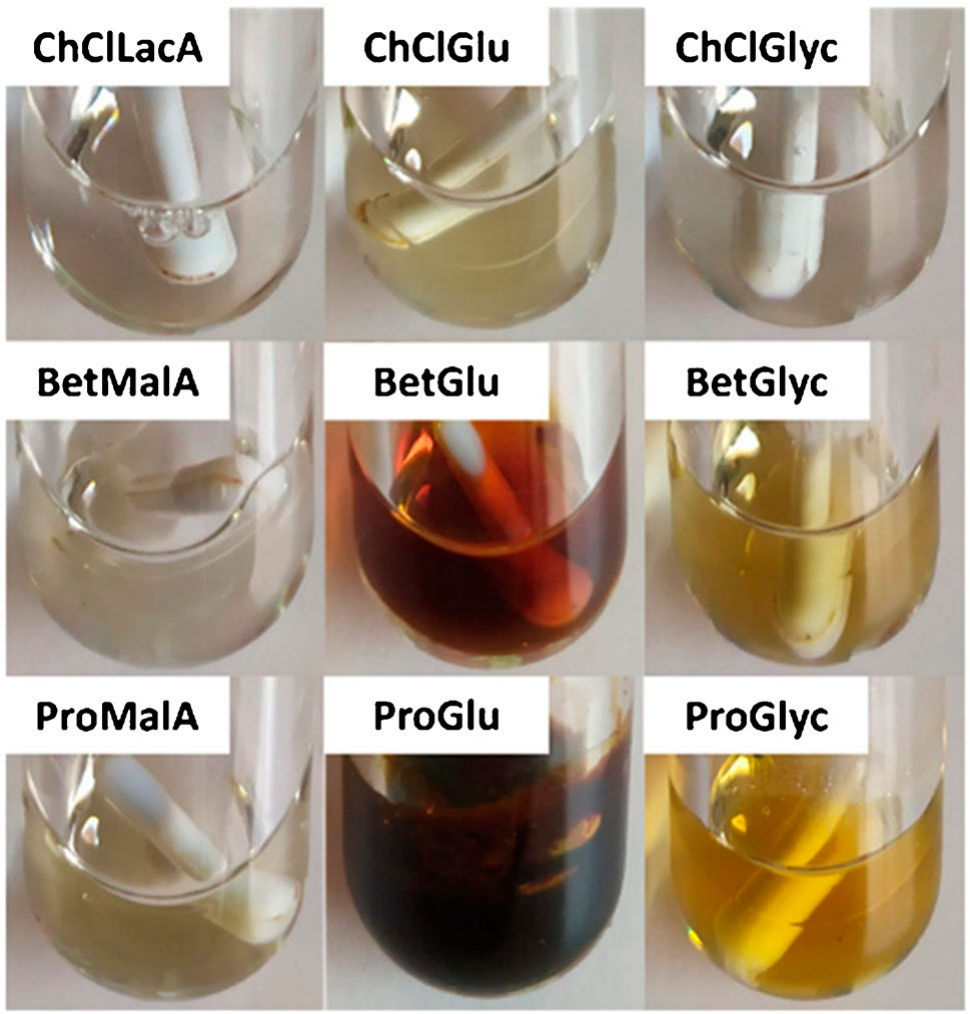
Extracts obtained after extraction by MAE at 130 °C for 10 min using different NADES as extracting solvents. Reproduced with permission from [32]

This point implies a great potential for further development. The need to avoid the use of petrochemically derived solvents is evident and the search for alternatives has been intense. In this regard, there are two main possibilities. The first one is characterized by the use of solvents from a bio-based origin. Bio-ethanol is the most used, as it can be relatively easily obtained by fermentation from a variety of natural biomasses. The use of ethanol together with advanced green extraction tools to recover bioactive compounds is quite extended [20]. In the last years, however, other bio-based solvents that may be obtained from renewable feedstock have been proposed in combination with green extraction methods including limonene [21], gamma-valerolactone [22], cyclopentyl methyl ether [23], or 2-methyl tetrahydrofuran [24]. Some of these solvents are particularly interesting to substitute low polarity volatile organic solvents, such as hexane, thanks to their physico-chemical properties [24]. The other group of alternative solvents is composed of natural deep eutectic solvents (NADES). Deep eutectic solvents emerged as greener alternatives to ionic liquids, and are formed by heating two components, a hydrogen bond acceptor and a hydrogen bond donor, that are solid separately but form a liquid after mixing and being combined. When the donor and acceptor are natural organic compounds, the mixture is called a NADES and presents similar properties but significantly less toxicity and enhanced biodegradability. An interesting feature is that NADES can be tailor-made with properties depending on their particular components. Although relatively new, the applications of NADES to a variety of extraction techniques with the aim to decrease the environmental impact of the recovery of natural bioactive compounds are constantly growing in the last years [25, 26]. There is a huge variety of different NADES reported that have been applied for the extraction of compounds of different chemical nature, such as polyphenols [27], carotenoids [28], and other pigments [29], saponins [30], or alkaloids [31], among others. Figure 2 shows how NADES selection significantly influences the recovery of proanthocyanidins from grape pomace [32], demonstrating the need for proper exhaustive study about the exact composition to be used for extraction. Interestingly, new hydrophobic NADES have been also identified, thanks to the study of Hansen solubility parameters, to defat soybeans during valorization of by-products, making them as useful alternatives to conventional organic solvents such as n-heptane [33].

### Reduce energy consumption using innovative technologies

Frequently, the information found in the literature about the greenness of an extraction process to obtain natural bioactives is limited to the type of solvent or the approach followed. However, other important parameters have to be considered to estimate if a process is more sustainable than its benchmark, and one of these is energy consumption and recovery. Although generally considered greener than conventional approaches, advanced extraction techniques sometimes require particular conditions that may involve energy-intensive processes, such as the use of very high pressure. For this reason, a proper assessment for each application could be of high interest. One of the most interesting tools in this sense is life cycle assessment (LCA). Thanks to the implementation of quantitative comparisons, the environmental impacts of each process and technology available can be actually compared. For instance, it has been shown how the use of microwaves- and ultrasound-assisted extractions is more energy-intensive per time unit than conventional processes such as maceration or Soxhlet extraction. However, when comparing a particular application, such as the recovery of the bioactive carotenoid astaxanthin from *Haematococcus pluvialis* microalga biomass, it is demonstrated how the use of MAE or UAE leads to lower environmental impacts than maceration, which needs less energy demands, thanks to an improved recovery and significantly lower processing time [34]. Indeed, the most efficient and faster methods may need less overall energy consumption per product unit, although the final result will strongly depend on the particular application. For instance, MAE was shown to require less energy than UAE for the recovery of phenolic acids from distillery by-products [35]. Other approaches to reduce energy consumption should be based on technological improvements on existing equipment, for instance through reusing the generated heat during extraction.

### Production of co-products instead of waste to include the bio- and agro-refining industry

**Fig. 3:**
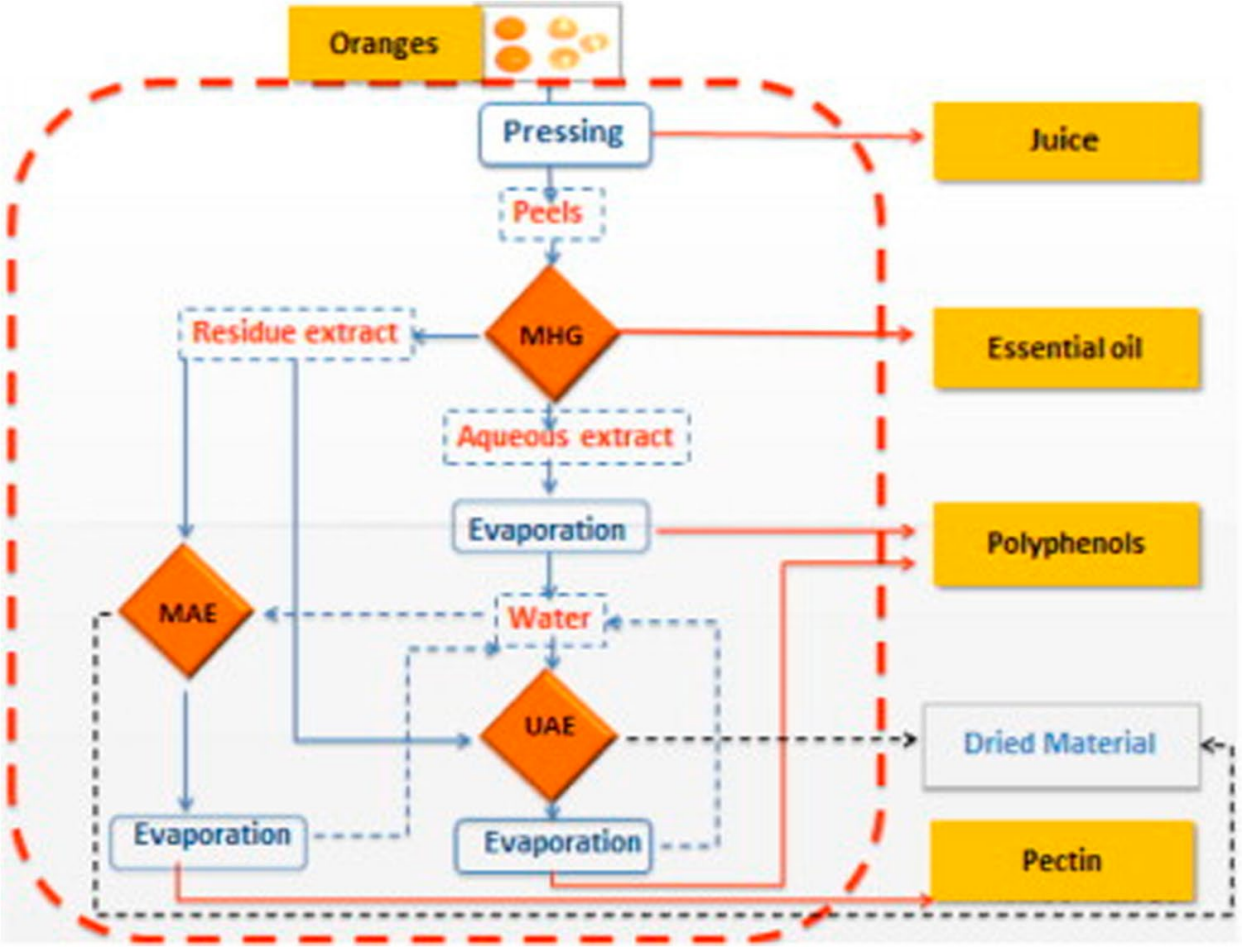
Scheme proposed for the biorefinery of orange peels waste, involving different extraction steps and the production of different added-value fractions. Reproduced with permission from [37]

The reduction of wastes that are meant to be disposed or landfilled through the generation of co-products is the most important aim of biorefinery approaches [36]. The target markets for biorefinery-derived products are wide, including food, pharmaceutical, cosmetic industries, and the generation of fine chemicals, among others. From a circular bioeconomy perspective, the concept and generation of biorefineries is highly relevant, as it may derive in complex and complete processes in which no real wastes are generated. For this reason, the implementation of this kind of approaches is a perfect match to green extraction techniques for the attainment of natural bioactive compounds, thus, increasing the overall sustainability and reducing the impact on the environment as much as possible. Several of these approaches have been already reported. Citrus peels retain a good potential for full valorization following a zero-waste approach, as they are still rich in several groups of components after juice extraction. Green approaches, based on the use of MAE and UAE, have been proposed to recover interesting natural bioactives, such as polyphenols [37]. Figure 3 shows the potential of a biorefinery implementation involving the use of green extraction processes for the valorization of orange peels. Similarly, a full biorefinery process involving the use of green extraction steps for the full valorization of *Saccharina latissima* seaweed biomass has been proposed, and its feasibility confirmed through a LCA studying all the relevant aspects, from cultivation and harvesting to the generation of different products for different fields [38]. The use of LCA allowed the identification of the most relevant environmental hot-spots that can be further improved. Similar approaches, adapted to each biomass, have been reported for other materials, such as legumes [39] and other vegetables [40, 41], halophytes [42], and microalgae. This latter group of organisms are promising biomasses for biorefinery approaches based both on their typical chemical compositions and to the possibility of using other waste streams as feeds for their production. As an illustrative example, a compressed fluids-based biorefinery-like approach was proposed to obtain different kinds of bioactives from *P. cruentum* biomass [43]. Each step was optimized separately and allowed combining tools with different polarity targets, such as SFE, PLE, and GXLs. Indeed, the use of compressed fluids-based extraction tools retains an excellent potential to be part of wider biorefinery processes [44].

### Reduce unit operations through technical innovation and favor safe, robust, and controlled processes

**Fig. 4:**
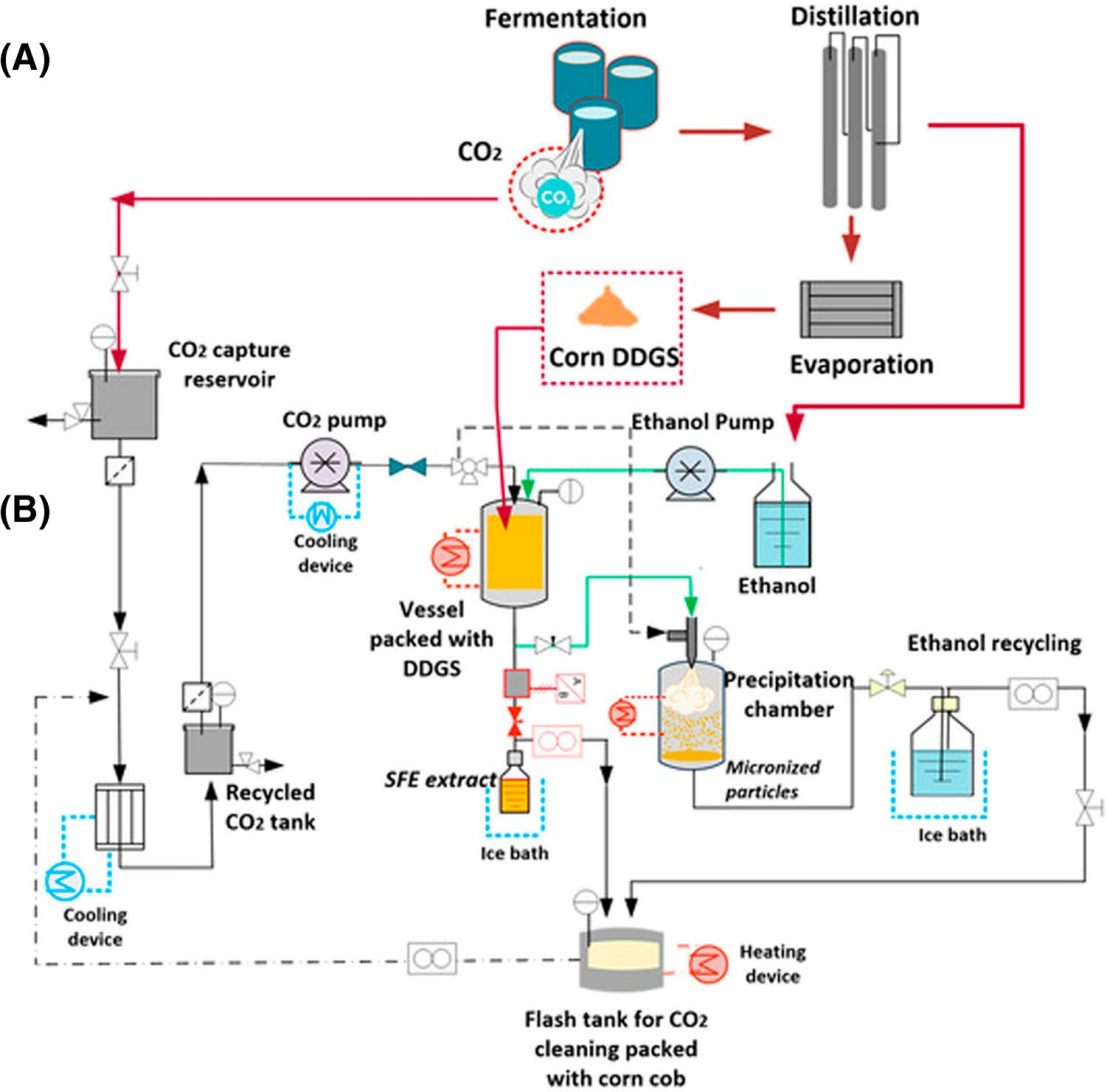
Diagram proposed for future directions on the optimization of dry grinding (**A**) via integration with supercritical technology (**B**) for the reutilization of corn by-products. Reproduced with permission from [45]

The reduction of unit operations is foreseen as a means to decrease the environmental impacts of the extraction processes. By implementing intensified processes, some steps can be avoided. An interesting proposal has been reported for the valorization of corn by-products, combining several approaches and technologies to reduce operating costs and to generate new products [45]. As can be observed in Fig. 4, a process strategy based on compressed fluids was designed to allow the recovery of different interesting products from corn milling by-products either by using supercritical fluids of pressurized ethanol. This contribution demonstrates the interest of using an equipment to operate under different conditions so that multiple products can be obtained in a single facility. Another example of process intensification is illustrated by the development of a one-pot strategy for the recovery of proteins and xanthohumol, an important bioactive compound, from spent hops from the brewing industry [46]. A DES-based extraction protocol was firstly developed to recover the interesting proteins and bioactives from the spent hops starting material. Later on, water is employed as an antisolvent to produce the precipitation of the target components, producing a selective recovery.

### Aim for a non-denatured and biodegradable extract without contaminants with “green” values

This last principle of green extraction of natural compounds involves and combines some of the research work already described in previous points. The generation of this kind of extracts is directly related to the solvents employed during extraction. A perfect example is the attainment of solvent-free extracts when using SFE with neat supercritical CO_2_ for the recovery of the bioactive compounds. Once the SFE process is finished and the pressure is released, the obtained extract is completely dried and free of solvent residues; this is the most favorable case. However, other interesting situations may occur when NADES are the selected extracting solvents. One of the problems that can be found towards the application of the obtained extracts is related to the need of evaporating or eliminating the extracting solvent. Thus, the search for the most appropriate NADES in a way that the extract can be straightforwardly used is of utmost interest [47]. In this case, aspects related to toxicity of the solvent should be considered, although it has also been pointed out the possibility that bioactive extracts are even more active when dissolved in a proper NADES than in other solvents [48]. Moreover, NADES have been shown to generally possess high stability at high temperatures and may also be used to increase the stability of the bioactive compounds over time [49].

The use of water as extracting solvent is, in principle, the greenest approach and provides with extracts that might be fully compatible for a given application. However, there are multiple examples of applications in which water has to be eliminated, which usually implies a costly process. In this case, the drying technique should be able to eliminate the water without significantly affecting the resulting extract. Another worrying point when extracting bioactive components with water is the chance of occurrence of non-desired reactions when high temperatures are used. This point has been repeatedly demonstrated and could raise safety concerns depending on the compounds formed in the extracts [50, 51].

## Outlook

As it is briefly illustrated in this manuscript, the field of green extraction of natural bioactive compounds is in constant evolution and increasing interest. It is foreseen that the future developments will expand the applicability of more sustainable extraction approaches for a variety of natural biomasses. In particular, the use of agri-food by-products as raw materials for the development of full valorization processes will most probably attract great attention, as the generation of circular bioeconomy processes is at the core of some of the most important research programs worldwide. Linked to this point, the use of novel bio-based safe and biodegradable solvents will continue its expansion. The use of solubility prediction models based on Hansen solubility parameters or conductor-like screening model for real solvents (COSMO-RS) can be helpful tools to develop this kind of new solvents, such as NADES, ad hoc for particular applications. However, apart from the starting biomasses and natural sources for bioactive components and solvents, some interesting aspects related to technology developments and implementations are also expected.

Up to now, as discussed here, the focus has been centered on the development of new applications and green processes to recover natural bioactives. However, as these techniques are gradually more widespread and routinely used, the chances for technology improvements are open. A particular aspect that should be tackled is related to the characteristic chemical variability of natural biomasses. As the outcome of the extraction procedure will be directly linked to the chemical composition of a particular natural sample, the need of fine-tuning or adjusting the extraction protocol or conditions to selectively recover the highest possible amount of the target compounds is a reality. In practice, this aspect implies that if the extraction conditions remain unchanged from batch to batch, the full utilization of the starting material may not be taking place. At this point is where digitalization technologies may provide a strong point. Automatic on-line tuning of the extraction process (i.e., extraction conditions) retains great promise to significantly improve the use of the raw materials, producing more valuable products with less environmental impact.

Moreover, technological improvements mean that extraction conditions that were not easily attainable before, can now be operated. An example of these future advancements is the use of ultra-high pressures under SFE. It is relatively frequent to find SFE processes operated at 400–600 bar, but the development of processes at significantly higher pressures, ca. 1000 bar, may change dramatically the achievable results. This kind of development opens the door for future complex developments in which new intensified processes may be designed for the full valorization of the natural biomass. In any case, it is also necessary to mention that, although much effort is being put today to study the possibilities behind numerous natural sources, the presented results are not always worthy. It is quite easy to find published reports in which green processes are developed for the recovery of valuable natural bioactive compounds from sources in which they are found in very little amount. Not every by-product, plant, or natural biomass can be effectively used or valorized to produce bioactives in a feasible way. Even if the developed process is, in principle, considered green, energy and other materials requirements will mean that the process is not economically favorable and, thus, other applications may be more promising.

Lastly, although a genuine environmental concern is present in this field, the risk to fall under pompous greenwashing speeches should be avoided. To this aim, the evaluation of the greenness of an extraction method is unavoidable. Close evaluation of the environmental impacts of the extraction processes should be offered to fully demonstrate their superiority compared to other possibilities, either by using LCA or any other measurement tool. Specific developments similar to AGREE tool [52] or green analytical procedure index (GAPI) to evaluate the greenness performance of analytical methods would be of great interest for the field of green extraction. The development of such dedicated tools for extraction processes and their widespread use will surely help researchers in the field to evaluate and assess the greenness performance of the developed process for each specific application.

## References

[1] Armstrong McKay DI, Staal A, Abrams JF, Winkelmann R, Sakschewski B, Loriani S, Fetzer I, Cornell SE, Rockström J, Lenton TM (2022). Exceeding 1.5°C global warming could trigger multiple climate tipping points. Science.

[2] Strand R, Kovacic Z, Funtowicz S, Benini L, Jesus A. Exiting the Anthropocene? Exploring fundamental change in our relationship with nature. European Environment Agency, Briefing no. 24/2022. 2022. 10.2800/37883

[3] Anastas PT, Warner JC (1998). Green chemistry: theory and practice.

[4] Armenta S, Garrigues S, de la Guardia M (2008). Green analytical chemistry. TrAC Trends Anal Chem.

[5] Gałuszka A, Migaszewski Z, Namieśnik J (2013). The 12 principles of green analytical chemistry and the SIGNIFICANCE mnemonic of green analytical practices. TrAC Trends Anal Chem.

[6] López-Lorente ÁI, Pena-Pereira F, Pedersen-Bjergaard S, Zuin VG, Ozkan SA, Psillakis E (2022). The ten principles of green sample preparation. TrAC Trends Anal Chem.

[7] Chemat F, Vian MA, Cravotto G (2012). Green extraction of natural products: concept and principles. Int J Mol Sci.

[8] Žlabur JŠ, Voća S, Brnč=ić M, Rimac-Brnč=ić S (2018). New trends in food technology for green recovery of bioactive compounds from plant materials. Role of materials science in food bioengineering.

[9] Berenguer CV, Andrade C, Pereira JAM, Perestrelo R, Câmara JS (2022). Current challenges in the sustainable valorisation of agri-food wastes: a review. Processes.

[10] Garcia-Perez P, Cassani L, Garcia-Oliveira P, Xiao J, Simal-Gandara J, Prieto MA, Lucini L (2023). Algal nutraceuticals: a perspective on metabolic diversity, current food applications, and prospects in the field of metabolomics. Food Chem.

[11] Yang Y, Hassan SHA, Awasthi MK, Gajendran B, Sharma M, Ji M-K, Salama E-S (2023). The recent progress on the bioactive compounds from algal biomass for human health applications. Food Biosci.

[12] Herrero M, Sánchez-Camargo del Pilar A, Cifuentes A, Ibáñez E (2015). Plants, seaweeds, microalgae and food by-products as natural sources of functional ingredients obtained using pressurized liquid extraction and supercritical fluid extraction. TrAC Trends Anal Chem.

[13] Barba FJ, Zhu Z, Koubaa M, Sant’Ana AS, Orlien V (2016). Green alternative methods for the extraction of antioxidant bioactive compounds from winery wastes and by-products: a review. Trends Food Sci Technol.

[14] Buvaneshwaran M, Radhakrishnan M, Natarajan V (2023). Influence of ultrasound-assisted extraction techniques on the valorization of agro-based industrial organic waste — a review. J Food Process Eng.

[15] Soares Mateus AR, Pena A, Sendón R, Almeida C, Nieto GA, Khwaldia K, Sanches Silva A (2023). By-products of dates, cherries, plums and artichokes: a source of valuable bioactive compounds. Trends Food Sci Technol.

[16] Pagliari S, Cannavacciuolo C, Celano R, Carabetta S, Russo M, Labra M, Campone L (2022). Valorisation, green extraction development, and metabolomic analysis of wild artichoke by-product using pressurised liquid extraction UPLC–HRMS and multivariate data analysis. Molecules.

[17] Molina AK, Gomes LC, Prieto MA, Ferreira IC, Pereira C, Dias MI, Barros L (2022). Extraction of chlorophylls from Daucus carota L. and Solanum lycopersicum var. cerasiforme crop by-products. Food Chem Adv.

[18] Georgiopoulou I, Louli V, Magoulas K (2023). Comparative study of conventional, microwave-assisted and supercritical fluid extraction of bioactive compounds from microalgae: the case of Scenedesmus obliquus. Separations.

[19] Vladić J, Jazić JM, Ferreira A, Maletić S, Cvetković D, Agbaba J, Vidović S, Gouveia L (2023). Application of green technology to extract clean and safe bioactive compounds from Tetradesmus obliquus biomass grown in poultry wastewater. Molecules.

[20] Hashemi B, Shiri F, Švec F, Nováková L (2022). Green solvents and approaches recently applied for extraction of natural bioactive compounds. TrAC Trends Anal Chem.

[21] Golmakani M-T, Mendiola JA, Rezaei K, Ibáñez E (2014). Pressurized limonene as an alternative bio-solvent for the extraction of lipids from marine microorganisms. J Supercrit Fluids.

[22] Silva SS, Justi M, Chagnoleau J-B, Papaiconomou N, Fernandez X, Santos SAO, Passos H, Ferreira AM, Coutinho JAP (2023). Using biobased solvents for the extraction of phenolic compounds from kiwifruit industry waste. Sep Purif Technol.

[23] Ozturk B, Winterburn J, Gonzalez-Miquel M (2019). Orange peel waste valorisation through limonene extraction using bio-based solvents. Biochem Eng J.

[24] Yara-Varón E, Fabiano-Tixier AS, Balcells M, Canela-Garayoa R, Bily A, Chemat F (2016). Is it possible to substitute hexane with green solvents for extraction of carotenoids? A theoretical versus experimental solubility study. RSC Adv.

[25] Cannavacciuolo C, Pagliari S, Frigerio J, Giustra CM, Labra M, Campone L (2022). Natural deep eutectic solvents (NADESs) combined with sustainable extraction techniques: a review of the green chemistry approach in food analysis. Foods.

[26] Meenu M, Bansal V, Rana S, Sharma N, Kumar V, Arora V, Garg M (2023). Deep eutectic solvents (DESs) and natural deep eutectic solvents (NADESs): designer solvents for green extraction of anthocyanin. Sus Chem Pharm.

[27] Santos-Martín M, Cubero-Cardoso J, González-Domínguez R, Cortés-Triviño E, Sayago A, Urbano J, Fernández-Recamales Á (2023). Ultrasound-assisted extraction of phenolic compounds from blueberry leaves using natural deep eutectic solvents (NADES) for the valorization of agrifood wastes. Biomass Bioenergy.

[28] Huang H, Zhu Y, Fu X, Zou Y, Li Q, Luo Z (2022). Integrated natural deep eutectic solvent and pulse-ultrasonication for efficient extraction of crocins from gardenia fruits (*Gardenia jasminoides* Ellis) and its bioactivities. Food Chem.

[29] Dai Y, Witkamp G-J, Verpoorte R, Choi YH (2013). Natural deep eutectic solvents as a new extraction media for phenolic metabolites in *Carthamus tinctorius* L. Anal Chem.

[30] Yang G-Y, Song J-N, Chang Y-Q, Wang L, Zheng Y-G, Zhang D, Guo L (2021). Natural deep eutectic solvents for the extraction of bioactive steroidal saponins from Dioscoreae Nipponicae Rhizoma. Molecules.

[31] Torres-Vega J, Gómez-Alonso S, Pérez-Navarro J, Pastene-Navarrete E (2020). Green extraction of alkaloids and polyphenols from *Peumus boldus* leaves with natural deep eutectic solvents and profiling by HPLC-PDA-IT-MS/MS and HPLC-QTOF-MS/MS. Plants.

[32] Neto RT, Santos SAO, Oliveira J, Silvestre AJD (2021). Impact of eutectic solvents utilization in the microwave assisted extraction of proanthocyanidins from grape pomace. Molecules.

[33] Bragagnolo FS, Socas-Rodríguez B, Mendiola JA, Cifuentes A, Funari CS, Ibáñez E (2022). Pressurized natural deep eutectic solvents: an alternative approach to agro-soy by-products. Front Nutr.

[34] Papadaki SG, Kyriakopoulou KE, Krokida MK (2016). Life cycle analysis of microalgae extraction techniques. Chem Eng Transact.

[35] Mikucka W, Zielińska M, Bułkowska K, Witońska I (2022). Valorization of distillery stillage by polyphenol recovery using microwave-assisted, ultrasound-assisted and conventional extractions. J Environ Manag.

[36] Cherubini F (2010). The biorefinery concept: using biomass instead of oil for producing energy and chemicals. Energy Convers Manag.

[37] Boukroufa M, Boutekedjiret C, Petigny L, Rakotomanomana N, Chemat F (2015). Bio-refinery of orange peels waste: a new concept based on integrated green and solvent free extraction processes using ultrasound and microwave techniques to obtain essential oil, polyphenols and pectin. Ultrason Sonochem.

[38] Nilsson AE, Bergman K, Gomez Barrio LP, Cabral EM, Tiwari BK (2022). Life cycle assessment of a seaweed-based biorefinery concept for production of food, materials, and energy. Algal Res.

[39] Tassoni A, Tedeschi T, Zurlini C, Cigognini IM, Petrusan J-I, Rodríguez Ó, Neri S, Celli A, Sisti L, Cinelli P, Signori F, Tsatsos G, Bondi M, Verstringe S, Bruggerman G, Corvini PFX (2020). State-of-the-art production chains for peas, beans and chickpeas—valorization of agro-industrial residues and applications of derived extracts. Molecules.

[40] Eslami E, Carpentieri S, Pataro G, Ferrari G (2022). A comprehensive overview of tomato processing by-product valorization by conventional methods versus emerging technologies. Foods.

[41] Da Silva J, De Brito ES, Ferreira SRS (2023). Biorefinery of cashew by-products: recovery of value-added compounds. Food Bioprocess Technol.

[42] Hulkko LSS, Rocha RM, Trentin R, Fredsgaard M, Chaturvedi T, Custódio L, Thomsen MH (2023). Bioactive extracts from Salicornia ramosissima J. Woods Biorefinery as a Source of Ingredients for High-Value Industries. Plants.

[43] Gallego R, Martínez M, Cifuentes A, Ibáñez E, Herrero M (2019). Development of a green downstream process for the valorization of *Porphyridium cruentum* biomass. Molecules.

[44] Herrero M, Ibañez E (2018). Green extraction processes, biorefineries and sustainability: recovery of high added-value products from natural sources. J SupercritFluids.

[45] Santana ÁL, Meireles MAA (2023). Valorization of cereal byproducts with supercritical technology: the case of corn. Processes.

[46] Grudniewska A, Pastyrczyk N (2022). New insight for spent hops utilization: simultaneous extraction of protein and xanthohumol using deep eutectic solvents. Biomass Conv Bioref.

[47] Li D (2022). Natural deep eutectic solvents in phytonutrient extraction and other applications. Front Plant Sci.

[48] Hikmawanti NPE, Ramadon D, Jantan I, Munim A (2021). Natural deep eutectic solvents (NADES): phytochemical extraction performance enhancer for pharmaceutical and nutraceutical product development. Plants.

[49] Dai Y, Verpoorte R, Choi YH (2014). Natural deep eutectic solvents providing enhanced stability of natural colorants from safflower (*Carthamus tinctorius*). Food Chem.

[50] Plaza M, Abrahamsson V, Turner C (2013). Extraction and neoformation of antioxidant compounds by pressurized hot water extraction from apple byproducts. J Agric Food Chem.

[51] Plaza M, Amigo-Benavent M, Del Castillo MD, Ibáñez E, Herrero M (2010). Facts about the formation of new antioxidants in natural samples after subcritical water extraction. Food Res Int.

[52] Pena-Pereira F, Wojnowski W, Tobiszewski M (2020). AGREE—analytical greenness metric approach and software. Anal Chem.

